# The Positive Effects of Cancer Survivor Support Service on Distress in South Korea: A Nationwide Prospective Study

**DOI:** 10.3389/fmed.2022.769221

**Published:** 2022-02-14

**Authors:** Hyun Jeong Lee, Young Ae Kim, Seong Yeob Ryu, Mison Chun, Chang-Yeol Yim, Hee-Taik Kang, Jung Hun Kang, Jung-Sik Huh, Jong-Heun Kim, Kyu-Hyoung Lim, So-Youn Jung, Hyoung-Cheol Kwon, Eurah Goh, Yeon-Seung Lee, Hee Young Ju, E. Hwa Yun, Yoon Jung Chang

**Affiliations:** ^1^Division of Cancer Control and Policy, National Cancer Center, Goyang, South Korea; ^2^National Cancer Survivorship Center, National Cancer Center, Goyang, South Korea; ^3^Department of Psychiatry and Behavioral Science, National Cancer Center, Goyang, South Korea; ^4^Department of Psychiatry, Seoul National University, Seoul, South Korea; ^5^Department of Surgery, Chonnam National University Medical School, Gwangju, South Korea; ^6^Department of Radiation Oncology, Ajou University School of Medicine, Suwon, South Korea; ^7^Department of Internal Medicine, Chonbuk National University Medical School, Research Institute of Clinical Medicine of Chonbuk National University, Jeonju, South Korea; ^8^Biomedical Research Institute of Chonbuk National University Hospital, Jeonju, South Korea; ^9^Department of Family Medicine, Chungbuk National University Hospital, Cheongju, South Korea; ^10^Department of Family Medicine, Chungbuk National University College of Medicine, Cheongju, South Korea; ^11^Division of Hematology-Oncology, Department of Internal Medicine, College of Medicine, Gyeong-Sang National University, Jinju, South Korea; ^12^Department of Medicine, Gyeongsang National University Hospital, Jinju, South Korea; ^13^Department of Urology, School of Medicine, Jeju National University, Jeju, South Korea; ^14^Department of Internal Medicine, Kangwon National University Hospital, Kangwon National University School of Medicine, Chuncheon, South Korea; ^15^Breast Cancer Center, National Cancer Center, Goyang, South Korea; ^16^Department of Radiation Oncology, Chonbuk National University Hospital, Jeonju, South Korea; ^17^Institute of Clinical Medicine of Chonbuk National University-Biomedical Research Institute, Chonbuk National University Hospital, Jeonju, South Korea; ^18^Department of Radiation Oncology, Chonbuk National University Medical School, Jeonju, South Korea; ^19^Department of Family Medicine, Postgraduate College of Medicine, Kangwon National University, Chuncheon, South Korea; ^20^Cancer Survivorship Branch, National Cancer Center, Goyang, South Korea; ^21^Department of Pediatrics, Samsung Medical Center, Seoul, South Korea; ^22^Division of Cancer Registration and Surveillance, National Cancer Center, Goyang, South Korea; ^23^Center for Cancer Prevention and Detection, National Cancer Center, Goyang, South Korea; ^24^National Hospice Center, National Cancer Center, Goyang, South Korea; ^25^Department of Cancer Control and Population Health, National Cancer Center Graduate School of Cancer Science and Policy, Goyang, South Korea

**Keywords:** cancer survivor, cancer survivorship management, distress, support service, government-led

## Abstract

**Background and Aim:**

Cancer survivors are gradually increasing, however, they suffer from various difficulties. We aimed to investigate the characteristics of cancer survivors and the effects of the services of the Korean Cancer Survivorship Center Pilot Project launched by the South Korean government on distress.

**Methods:**

A prospective observational cohort study was performed on cancer survivors who completed primary treatment. Cancer survivors' distress and symptoms such as fatigue, pain, depressive mood, anxiety, and insomnia were evaluated by well-trained nurses. Regarding their needs, medical and psychosocial support services were provided.

**Results:**

This study included 1,921 cancer survivors, with a mean age of 57.3 years (68.7% females). Breast cancer was most common, followed by stomach and colorectal cancer. Psychosocial and medical support decreased the percentage of the high-distress group from 50.9 to 30.5% and decreased the percentage of cancer survivors with high scores in fatigue, pain, anxiety, depressive mood, and insomnia. The independent predictors of a low distress level after the use of the services were older age, the relief of fatigue, pain, and insomnia.

**Conclusion:**

This study showed that psychosocial and medical support is associated with the lower distress and physical and mental symptoms of cancer survivors. Psychosocial and medical support could contribute to distress relief in cancer survivors. Further management strategies for fatigue, pain and insomnia are required.

## Introduction

The numbers of cancer survivors are gradually increasing owing to advances in screening and treatment strategies. This has resulted in a growing interest in the quality of life (QOL) of cancer survivors. However, they still suffer from various difficulties including long-term treatment effects, late-effects of treatment, distress, anxiety, uncertainty, fatigue, secondary malignancy, and other medical illness (1–6). Unmanaged distress has negative effects on all-cause and cancer-related morbidity and mortality, as well as quality of life (7–9). Distress is defined as ‘an unpleasant experience of a mental, physical, social, or spiritual nature' (10). Because distress is caused by physical, psychological, and social problems, psychosocial and medical support should be included in cancer care (11).

The 5-year relative survival rate of cancer patients in South Korea improved substantially from 41.2% for the period 1993–1995 to 70.6% for the period 2012–2016; there were approximately 1,740,000 cancer survivors in 2016 (12). The age-standardized 5-year net survival rates in South Korea are among the highest of the 32 countries in the Organization for Economic Cooperation and Development (OECD); 5-year survival rates are 71.8% in colon cancer patients, 71.1% in rectal cancer patients, 68.9% in stomach cancer patients (ranked first among OECD countries) and 25.1% in lung cancer patients (ranked third among OECD countries) (13). Nevertheless, South Korea has lacked a systematic support system for cancer survivors. To improve the health status of cancer survivors and facilitate their return to normal social lives, the Korean Ministry of Health and Welfare together with the National Cancer Center, a government-endorsed organization, launched the Korean Cancer Survivorship Center Pilot Project (K-CSCP) in July 2017.

We investigated characteristics of cancer survivors visiting cancer survivorship centers (CSCs) and the associations of the Korean Cancer Survivorship Center Pilot Program with survivors' distress and distress-related symptoms including pain, fatigue, anxiety, depressive mood, and insomnia.

## Materials and Methods

### Study Design

This study was a prospective, observational cohort study performed from July 2017 to December 2018 in South Korea. Seven regional cancer centers and one national cancer center covering the regional areas in South Korea were designated in 2017, and one more center was joined in 2018.

### Participants

This program was targeted to any adult cancer survivor who completed curative-intent anti-cancer treatment including surgery, chemotherapy, or radiotherapy. Survivors undergoing maintenance hormonal therapy were also included. This study excluded individuals at the end-stage of cancer needing hospice-palliative care. Cancer survivors were recruited through a variety of channels, such as referrals from medical staff, recommendations from other patients, or advertisements. A total of 2,601 cancer survivors registered to the CSCs, however, 156 individuals refused to participate in this study; therefore, 2,445 subjects were finally included. Informed consent was obtained from each study participant. The study was approved by the institutional review boards of National Cancer Center (IRB No.: NCC2017-0204) and other seven hospitals.

### Outcome Measures

Tools included physical and mental health, and social welfare items. The baseline assessment tool comprised a self-report questionnaire and an observer-assessment tool used by nurses. We gathered data about basic sociodemographic factors (age, sex, education level, and marital status), income, life behavior (smoking and drinking habits), health conditions (body mass index, comorbidity), distress, and distress related symptoms including anxiety, depression, insomnia, fatigue, and pain by paper questionnaire and interview. We used patient treatment summaries to obtain clinical information related to cancer diagnosis and treatment.

We used the National Comprehensive Cancer Network (NCCN) distress thermometer (DT) and Problem list for distress screening, which were validated in Korean (14, 15). The DT was a one-item measure of distress—on a scale of zero to 10, 10 being the worst. A cut-off of ≥4 has been accepted by the NCCN to indicate clinically meaningful distress (10). Using this cut-off, cancer survivors were divided into high- and low-distress groups. For screening other domain including fatigue, pain, anxiety, depressive mood, and insomnia, we used 11-point (0–10) Likert scales (16, 17). We used the EuroQuo-visual analog scale (EQ-VAS) to assess the quality of life (18, 19).

### Study Procedure

The CSC nurses who were oncology nurses or nurses with at least 2-year experience in cancer care conducted individual education or counseling during the baseline assessment. The CSC social workers were medical social workers or social workers with at least 2-year experience in counseling in other institutions. Well-trained nurses evaluate cancer survivors in various areas including distress, distress related symptoms, and social welfare area. After the initial assessment, survivors were divided into the low-risk groups (all areas with low symptom burden) and the high-risk group (one or more of the eight areas with moderate to high symptom burden). Cancer survivors in the high-risk group were evaluated by doctors at CSC's cancer-survivorship clinic because of their greater symptom burdens and unmet needs for supportive care and referred to specialists in other medical departments as needed.

Then, they were provided with cancer survivor support services (Supplementary Table 1) as needed. When the assessment score was 4 or more, we provided services by symptom categories. A multidisciplinary cancer survivor support team comprising doctors, nurses, and social workers provided the cancer survivor support services. Primarily several educational programs of various topics, including management of adverse effects of cancer treatment, proper nutrition, undergoing secondary malignancy screening, the importance of vaccinations, and maintaining a healthy lifestyle such as quitting smoking or alcohol, managing distress, and ensuring good sleep routines were provided. In addition, counseling, group programs such as programs of exercise and psychological support, social welfare counseling or cancer survivorship clinics treatment were provided if needed. The composition of the program differed according to the conditions in each CSC.

They were reassessed after 1 or 3 months from baseline according to their risk levels using part of the initial assessment tool. Of 2,445 subjects, 1,921 subjects completed re-assessment of distress and were included in the analyses.

### Statistical Analyses

We analyzed the continuous variables using an independent *t*-test, and categorical variables using the chi-square test (or Fisher's exact test, if appropriate). We used the McNemar test to evaluate the changes in proportion of distress, and distress related symptoms including fatigue, pain, anxiety, depressive mood, and insomnia. Multivariate analysis of covariance was used to examine estimated mean scores in EQ-VAS, distress, pain, fatigue, anxiety, depressive mood, and insomnia according to distress levels after adjusting for age, sex, education levels, economic status, cancer diagnosis, cancer treatment, and time from diagnosis to CSC visit. Univariate logistic regression was carried out, and significant variables were entered into multivariate logistic regression to determine predictors of a low-distress level after services. Statistical analyses were performed using SAS (version 9.4, SAS Inc., Cary, NC, USA). A two-sided *p*-value < 0.05 was considered to indicate significance.

## Results

### Baseline Characteristics

This study included 1,921 cancer survivors, with a mean age of 57.3 years (68.7% females). The most common cancer was breast cancer (41.3%), followed by stomach cancer (21.7%), colorectal cancer (11.5%), and other cancers. Cancer survivors were grouped by their DT scores; 49.1% had low distress (DT score < 4) and 50.9% had high distress (DT score ≥ 4). Compared to the low-distress group, the high-distress group were younger and had a higher percentage of the high-risk groups in terms of symptom burdens at baseline, being female, having breast cancer, and undergoing non-surgical treatment modalities (chemotherapy, radiotherapy, or other anti-cancer treatment). Conversely, the high-distress group had a lower percentage of patients with stomach or colorectal cancers, compared to the low-distress group (Table 1).

**Table 1:** Characteristics of cancer survivors according to distress level at baseline.

**Variables**	**Total**		**Low-distress group** ^**†**^		**High-distress group** ^**‡**^		***p*-value**
	***n*** **= 1,921**		***n*** **= 943 (49.1%)**		***n*** **= 978 (50.9%)**		
	***n***	**(%)**	***n***	**(%)**	***n***	**(%)**	
Age, year	57.3 ± 11.2		58.8 ± 10.9		55.9 ± 11.2		<0.001^**^
Female	1,320	(68.7)	603	(63.9)	717	(73.3)	<0.001^**^
Education							
< High school	563	(29.3)	294	(31.2)	269	(27.5)	0.077
High school	702	(36.5)	342	(36.3)	360	(36.8)	0.805
>High school	635	(33.1)	299	(31.7)	336	(34.4)	0.217
Income, dollars/month							
<850	395	(21.9)	183	(20.7)	212	(23.0)	0.244
850–1,700	319	(16.6)	154	(16.3)	165	(16.9)	0.750
1,700–2,550	333	(17.3)	163	(17.3)	170	(17.4)	0.955
2,550–3,400	304	(15.8)	160	(17.0)	144	(14.7)	0.178
≥3,400	456	(23.7)	224	(23.8)	232	(23.7)	0.987
Married/living with partner	1,577	(82.9)	789	(84.2)	788	(81.6)	0.128
With jobs	644	(33.6)	329	(34.9)	315	(32.4)	0.244
Time from cancer diagnosis, years	3.4 ± 3.7		3.6 ± 4.1		3.1 ± 3.3		0.003^*^
Cancer diagnosis							
Breast cancer	792	(41.3)	359	(38.2)	433	(44.4)	0.005^*^
Stomach cancer	415	(21.7)	227	(24.1)	188	(19.3)	0.010^*^
Colorectal cancer	221	(11.5)	124	(13.2)	97	(9.9)	0.027^*^
Other cancer	488	(25.5)	231	(24.5)	257	(26.4)	0.363
Cancer treatment							
Surgery	1,816	(95.8)	890	(95.4)	926	(96.2)	0.406
Chemotherapy	973	(51.3)	454	(48.7)	519	(54.0)	0.021^*^
Radiotherapy	895	(47.2)	411	(44.1)	484	(50.3)	0.007^*^
Other cancer treatment (hormone therapy, etc.)	515	(27.4)	223	(24.1)	292	(30.6)	0.002^*^
CSC visit route							
Recommendation of hospital staff	639	(33.3)	358	(38.0)	281	(28.7)	<0.001^**^
Recommendation of oncologist	365	(19.0)	210	(22.3)	155	(15.8)	<0.001^**^
In-hospital advertising (poster, leaflet, banner, etc.)	339	(17.6)	117	(12.4)	222	(22.7)	<0.001^**^
Recommendation from other patients	159	(8.3)	68	(7.2)	91	(9.3)	0.096
Recommendation from family and friends	77	(4.0)	38	(4.0)	39	(4.0)	0.963
Advertising media	36	(1.9)	18	(1.9)	18	(1.8)	0.912
Other	136	(7.1)	68	(7.2)	68	(7.0)	0.826
Scores at baseline							
EQ-VAS	67.4 ± 18.0		72.8 ± 16.9		62.1 ± 17.5		<0.001^**^
Distress	4.0 ± 2.5		1.9 ± 1.1		6.0 ± 1.6		<0.001^**^
Fatigue	4.2 ± 2.6		3.1 ± 2.2		5.3 ± 2.5		<0.001^**^
Pain	2.4 ± 2.7		1.6 ± 2.1		3.2 ± 2.9		<0.001^**^
Anxiety	2.8 ± 2.7		1.5 ± 1.9		4.0 ± 2.8		<0.001^**^
Depressive mood	2.5 ± 2.7		1.1 ± 1.6		3.8 ± 2.9		<0.001^**^
Insomnia	2.8 ± 2.9		1.8 ± 2.3		3.8 ± 3.1		<0.001^**^
High-risk group^§^	1,475	(76.8)	622	(66.0)	853	(87.2)	<0.001^**^

† *Low-distress group had distress thermometer (DT) scores <4*.

‡ *High-distress group had DT scores ≥ 4*.

§ *The high-risk group had moderate to high symptom burdens in one or more areas among eight areas (nutrition and diet, fatigue, pain, anxiety, depressive mood, insomnia, exercise, and social welfare)*.

* *p-value < 0.05*.

** *p-value < 0.001*.

*CSC, Cancer Survivorship Center*.

### Changes of Proportion of Distress and Distress Related Symptoms After Using the Cancer Survivor Support Services

**Figure 1:**
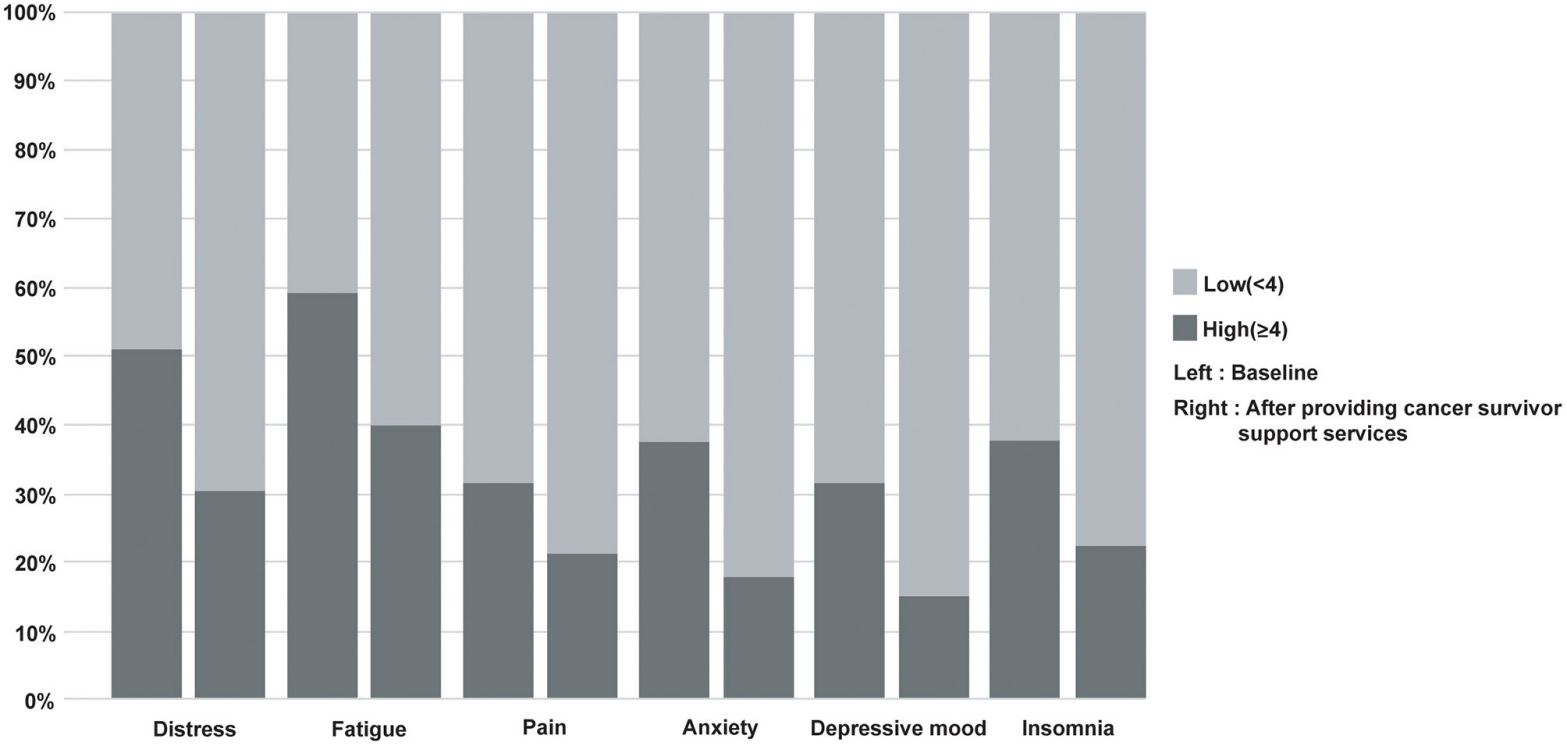
Changes in the proportion of high scores in distress, fatigue, pain, anxiety, depressive mood, and insomnia after providing support services for cancer survivors. A McNemar test was performed. All *p* < 0.001.

Cancer survivors receiving support services decreased the proportion of people with high symptoms scores (4 point or more), such as distress (978, 50.9% vs. 586, 30.5%), fatigue (1,078, 59.2% vs. 726, 39.8%), pain (567, 31.6% vs. 380, 21.2%), anxiety (678, 37.5% vs. 322, 17.8%), depressive mood (571, 31.7% vs. 275 15.2%), and insomnia (682, 37.6% vs. 409, 22.5%), as assessed after 1 or 3 months compared to baseline (all *p* < 0.001) (Figure 1).

### Characteristics of Cancer Survivors According to Distress Levels After Using the Cancer Survivor Support Services

Table 2 shows demographic and clinical characteristics in low- and high-distress group after cancer survivor support services. Compared with the low-distress group after receiving the service, the high-distress group was younger, the proportion of male, those with a monthly income of 2,550–3,400 dollars, those with high school graduates and below were low, and the proportion of those with a monthly income of ≥3,400 dollars was high. The proportion of stomach and colorectal cancer survivors in the low-distress group was higher. The proportion of breast cancer survivors was higher in the high-distress group, and the proportions of survivors who received chemotherapy, radiotherapy, and other cancer treatment (e.g., hormonal therapy, targeted therapy, endoscopic mucosal resection, radioactive iodine therapy, etc.) was higher. The high-distress group had lower quality of life and higher fatigue, pain, anxiety, depressive mood, and insomnia than the low-distress group, after adjusting for age, sex, education levels, economic status, cancer diagnosis, cancer treatment and time from diagnosis to visit.

**Table 2:** Characteristics of cancer survivors according to distress levels after using cancer survivor support services.

	**Low-distress group** ^**†**^		**High-distress group** ^**‡**^		***p*-value**
	***n*** **= 1,335 (69.5%)**		***n*** **= 586 (30.5%)**		
	***n***	**(%)**	***n***	**(%)**	
Age, year	58.3 ± 11.0		55.0 ± 11.1		<0.001^**^
Female	872	(65.3)	448	(76.5)	<0.001^**^
Education					
< High school	414	(31.0)	149	(25.4)	0.013^*^
High school	484	(36.3)	218	(37.2)	0.692
>High school	424	(31.8)	211	(36.0)	0.068
Income, dollars/month					
<850	277	(22.0)	118	(21.5)	0.783
850–1,700	219	(16.4)	100	(17.1)	0.720
1,700–2,550	231	(17.3)	102	(17.4)	0.956
2,550–3,400	230	(17.2)	74	(12.6)	0.011^*^
≥3,400	300	(22.5)	156	(26.6)	0.049^*^
Married/living with partner	1,105	(83.4)	472	(81.7)	0.356
With job	466	(34.9)	178	(30.6)	0.064
Time from cancer diagnosis, year	3.5 ± 3.8		3.1 ± 3.4		0.015^*^
Cancer diagnosis					
Breast cancer	504	(37.9)	288	(49.2)	<0.001^**^
Stomach cancer	330	(24.8)	85	(14.5)	<0.001^**^
Colorectal cancer	170	(12.8)	51	(8.7)	0.011^*^
Other cancer	327	(24.6)	161	(27.5)	0.172
Cancer treatment					
Surgery	1,267	(96.3)	549	(94.5)	0.064
Chemotherapy	645	(49.0)	328	(56.6)	0.003^*^
Radiotherapy	570	(43.4)	325	(55.8)	<0.001^**^
Other cancer treatment	323	(24.7)	192	(33.5)	<0.001^**^
Scores after services^§^					
EQ-VAS	78.7 ± 0.9		66.9 ± 1.0		<0.001^**^
Distress	1.8 ± 0.1		5.1 ± 0.1		<0.001^**^
Fatigue	2.7 ± 0.1		4.8 ± 0.1		<0.001^**^
Pain	1.1 ± 0.1		3.0 ± 0.2		<0.001^**^
Anxiety	1.3 ± 0.1		3.2 ± 0.2		<0.001^**^
Depressive mood	1.3 ± 0.1		3.1 ± 0.1		<0.001^**^
Insomnia	0.9 ± 0.1		2.8 ± 0.1		<0.001^**^

† *Low-distress group had distress thermometer (DT) score < 4*.

‡ *High-distress group had DT scores ≥ 4*.

§ *MANCOVA, estimated mean ± standard error; adjusted for age, sex, education levels, economic status, cancer diagnosis, cancer treatment and time from diagnosis to visit*.

* *p-value < 0.05*.

** *p-value < 0.001*.

*EQ-VAS, EuroQuo-visual analog scale*.

### Predictors of Low Distress Level After Using the Cancer Survivor Support Services

Univariate analyses revealed that older age, male, a lower education level, longer length of time from cancer diagnosis, stomach, and colorectal cancer (vs. breast cancer), no history of chemotherapy, radiotherapy, or other anti-cancer treatment, and improvement of fatigue, pain, anxiety, and insomnia were associated with a low distress level after services. Multivariate logistic regression analysis showed that after using services, older age and improvement of fatigue, pain, and insomnia were independently associated with a low distress level (Table 3).

**Table 3:** Predicting factors of a low distress level after services.

**Cancer-survivor characteristics**		**Univariate analysis**			**Multivariate analysis**		
		**OR**	**95% CI**	***p*-value**	**OR**	**95% CI**	***p*-value**
Age, year		1.028	1.019–1.037	<0.001^**^	1.023	1.010–1.037	<0.001^**^
Female (ref = male)		0.580	0.465–0.724	<0.001^**^	0.825	0.598–1.137	0.240
Education level		0.853	0.754–0.965	0.012^*^	1.042	0.888–1.222	0.615
Income		0.980	0.916–1.048	0.550			
Cancer survivors with jobs (ref = without jobs)		1.219	0.989–1.502	0.064			
Married/living with partner (ref = single/divorced, widowed)		1.128	0.874–1.456	0.356			
Time from cancer diagnosis, year		1.036	1.007–1.065	0.016^*^	0.995	0.964–1.027	0.760
Cancer diagnosis	Breast cancer	Ref			Ref		
	Stomach cancer	2.218	1.679–2.932	<0.001^*^	1.235	0.775–1.967	0.374
	Colorectal cancer	1.905	1.349–2.689	<0.001^*^	1.183	0.736–1.900	0.487
	Other cancer	1.161	0.915–1.472	0.220	0.811	0.573–1.149	0.239
Cancer treatment	Surgery (ref = no surgery)	1.539	0.973–2.433	0.065			
	Chemotherapy (ref = no chemotherapy)	0.740	0.608–0.900	0.003^*^	0.796	0.627–1.010	0.060
	Radiotherapy (ref = no radiotherapy)	0.606	0.498–0.737	<0.001^*^	0.846	0.624–1.148	0.284
	Other treatment (ref = no other treatment)	0.650	0.525–0.805	<0.001^*^	0.898	0.685–1.178	0.437
Change of scores from baseline	ΔFatigue	1.163	1.109–1.220	<0.001^*^	1.124	1.064–1.187	<0.001^**^
	ΔPain	1.122	1.066–1.181	<0.001^*^	1.065	1.004–1.129	0.036^*^
	ΔAnxiety	1.054	1.005–1.104	0.029^*^	0.996	0.943–1.052	0.881
	ΔDepressive mood	1.039	0.991–1.089	0.114			
	ΔInsomnia	1.112	1.063–1.164	<0.001^*^	1.079	1.024–1.137	0.004^*^

* *p-value < 0.05*.

** *p-value < 0.001*.

*OR, Odds ratio; CI, confidence interval; ref, reference*.

## Discussion

This study showed that using the cancer survivor support services was related to amelioration of the distress and symptoms such as fatigue, pain, anxiety, depressive mood, and insomnia in cancer survivors. Our multidisciplinary cancer survivor support services include education, counseling, programs of exercise, or psychological support and cancer survivorship clinics, if needed. Previous studies showed that psychoeducational intervention, exercise, and education programs could improve symptom clusters including fatigue, depression, insomnia, pain, functional performance, and quality of life (20, 21).

Within this study population, 75% were stomach, colorectal or breast cancer survivors, reflecting the current cancer epidemiological profile and clinical outcomes. In South Korea, the most common malignancy is stomach cancer (13.3%), followed by colorectal cancer (12.3%); breast cancer is the fifth most common cancer (9.5%) in the entire population and the most common cancer among females (19.9%). Furthermore, these three cancers have high 5-year survival rates (76.0, 75.9, and 92.7%, respectively) (12).

Younger cancer survivors experienced more distress levels at baseline and were not likely to improve the distress after using cancer survivor support services. A previous study (22) implied that younger patients may suffer a higher level of distress (22), perhaps due to economic vulnerability from decreased income, the larger disruption of social and familial roles in earlier developmental stages (23), a higher fear of progression, and more aggressive anti-cancer treatments (24). In a previous study, younger survivors also reported more pain impact and intensity than older survivors (25).

At baseline, the high-distress group had high female predominance, with breast cancer survivors accounting for about half of this population. The need for supportive care among breast cancer survivors is relatively high (26). Chemotherapy, radiotherapy, and long-term hormone therapy are widely applied to reduce the risks of breast cancer recurrence (27). In particular, the many physical and mental symptoms caused by hormone therapy, e.g., hot flushes, night sweats, arthralgia, osteoporosis, insomnia, and depression (28), may lead to higher distress in survivors.

Older age, greater improvement of fatigue, pain and insomnia were independently associated with a low distress level after using the services in this study. Since our services focused on the general problem of cancer survivors and most cancer survivors visiting the CSC were middle-aged or old, specialized services for young cancer survivors are lacking. Therefore, they were unlikely to improve their distress after using support services.

Cancer-related fatigue is a common and distressing adverse effect of cancer and cancer therapy (29, 30); fatigue is a common barrier for returning to work and the activities of daily life (31, 32). Fatigue and distress have reciprocal effects on each other (33). A study of prostate cancer survivors showed that the risk of distress was positively associated with fatigue, insomnia, urinary, bowel and androgen deprivation therapy-related symptoms, and financial difficulties (34). Pain is also a common problem in cancer survivors (6). Insomnia is related to fatigue itself. Insomnia contributes to additional risk for persistent fatigue after cancer treatment (35). However, from a recent study (36), cognitive behavioral therapy did not significantly improve fatigue, although it could improve sleep.

Our project has several strengths. First, to the best of our knowledge, this pilot project is the first government-led national cancer survivor management program with adequate follow-up, worldwide. Our cancer survivorship management program is government-funded and based in academic cancer centers that were designated as regional cancer centers by the government to reduce the regional inequality of cancer care. South Koreans have good access to medical services. However, due to the lack of support systems for health and social welfare beyond the medical treatment of cancer survivors, this project was designed to develop and disseminate national programs, and to develop and operate services according to regional characteristics. Several countries have other cancer survivorship management models. For example, cancer survivorship management in the United States of America is conducted mainly by academic cancer centers. In the United Kingdom, the survivorship care model is based on the country-wide healthcare system, although medical care and other supportive services are provided by community-based primary-care clinics, private institutions, or cancer charities (37). In Australia, medical care and other supportive services for cancer survivors are provided by cancer centers in public hospitals, in part supported by the state government and private foundation. Their supportive services include providing information to cancer survivors and training for healthcare professionals (38). Compared to previous studies, the advantage of our study was that it provided integrated support services to cancer survivors that manage various biopsychosocial issues and include psychosocial and medical support.

This project had several limitations. First, selection bias might have occurred, in terms that those who were unable to visit a center during the daytime could not participate in this project. Second, the intervention services were diverse according to centers, and not standardized, making a quantitative assessment of their efficacies difficult. Further study designs should include standardized services. Third, there is a limitation as our assessment tool of distress-related symptoms was simple, not a comprehensive evaluation. However, it has the advantage of being easy to use and easy to spread to a large population. Fourth, primary due to lack of control group, our study result might be interpreted, considering the possibility that some people might experience self-remission even without any interventions. Because the psychologists were not mandatory team member, there were many CSCs without psychologists. This absence of psychologists could be a limitation of our psychological interventions. Further study would also be valuable to include spiritual intervention.

## Conclusion

This study is the first to report a nationwide government-led cancer survivor management program. Our pilot study showed positive effects on physical and mental health in cancer survivors. Predictors of a low-distress level after utilization of services were older age, and greater improvement in symptoms of fatigue, pain, and insomnia. Specialized support services to manage major issues for younger cancer survivors, i.e., childbearing, childcare, marriage, study, or career, are needed. Furthermore, management strategies to control fatigue, pain and insomnia are also required.

## Data Availability Statement

The raw data supporting the conclusions of this article will be made available by the authors, without undue reservation.

## Ethics Statement

The studies involving human participants were reviewed and approved by the Institutional Review Boards of National Cancer Center (IRB No.: NCC2017-0204). The patients/participants provided their written informed consent to participate in this study.

## Author Contributions

HJL and YAK: conceptualization and writing—review and editing. HJL, YAK, and HYJ: methodology. HJL: formal analysis and writing—original draft preparation. SYR, MC, C-YY, H-TK, JHK, J-SH, J-HK, K-HL, S-YJ, H-CK, EG, Y-SL, and HJL: data curation. YJC: supervision. YAK and YJC: project administration. All authors contributed to the article and approved the submitted version.

## Conflict of Interest

The authors declare that the research was conducted in the absence of any commercial or financial relationships that could be construed as a potential conflict of interest.

## Publisher's Note

All claims expressed in this article are solely those of the authors and do not necessarily represent those of their affiliated organizations, or those of the publisher, the editors and the reviewers. Any product that may be evaluated in this article, or claim that may be made by its manufacturer, is not guaranteed or endorsed by the publisher.
