# Supplementary material for: The Positive Effects of Cancer Survivor Support Service on Distress in South Korea: A Nationwide Prospective Study
Source: Front Med (Lausanne). 2022 Feb 14;9:769221. doi: 10.3389/fmed.2022.769221 (PMC8882982; doi:10.3389/fmed.2022.769221)
Supplement: Supplementary file 1 [file Data_Sheet_1.docx]

**Supplementary**

**Korean Cancer Survivorship Center Pilot Project (K-CSCP)**

K-CSCP is a government-led project in South Korea that is to improve the health status of cancer survivors and facilitate their return to normal social lives since July 2017. In the beginning, there were seven cancer survivorship centers (CSCs) at the Hwasun-gun (Gwangju∙Jeonnam CSC), Chuncheon-si (Gangwon CSC), Cheongju-si (Chungbuk CSC), Jinju-si (Gyeongnam CSC), Jeju-si (Jeju CSC), and Goyang-si (National Cancer Center CSC). In 2018, one CSC was added at the Suwon-si (Gyeonggi CSC). Each center gets paid budget by the government and local government. The government and local governments share the same proportion of the budget (50%: 50%).

CSCs must have doctors as head of CSC or clinic doctors, nurses, and a social worker. The CSC nurses who were oncology nurses or nurses with at least 2-year experience in cancer care conducted individual education or counseling during the baseline assessment. The CSC social workers were medical social workers or social workers with at least 2-year experience in counseling in other institutions.

When cancer survivors visit the CSC, after obtaining consent, the initial assessment is performed by the CSC nurse. The initial assessment includes distress, distress-related symptoms, and social welfare area. By the result of an assessment, cancer survivors were divided into the low-risk group (all areas with low symptom burden) and the high-risk group (one or more of the eight areas with moderate to high symptom burden). Cancer survivors in the high-risk group were evaluated by doctors at the CSC’s cancer survivorship clinic. After evaluation, the doctor writes a cancer survivor support plan.

Cancer survivors were provided with cancer survivor support services (Supplementary Table 1) as needed. If their symptom score was 4 or more, we provided services. The educational programs were primarily provided. The topics of educational programs were the management of adverse effects of cancer treatment, proper nutrition, undergoing secondary malignancy screening, the importance of vaccinations, and maintaining a healthy lifestyle such as quitting smoking or alcohol, managing distress, and ensuring good sleep routines. Counseling, group programs such as programs of exercise and psychological support, social welfare counseling, or cancer survivorship clinics treatment were provided if needed. The educational programs and counseling were mainly provided by CSC nurses, doctors, or social workers. Some programs of exercise and psychological support programs were run by external instructors. The composition of the program differed according to the conditions in each CSC.

Cancer survivors in the low-risk group were reassessed after 1 month and those in the high-risk group were reassessed after 1 month and 3 months from baseline. The assessment tool was used as part of the initial assessment tool.

**Supplementary table 1. Cancer Survivor Support Services**

| Category | Cancer Survivor Support Programs |
| --- | --- |
| Physical Health Management | Education of cancer-related fatigue |
|  | Exercise program |
|  | Pain management program |
| Mental Health Management | Education of distress |
|  | Education of communication skill |
|  | Group counseling |
|  | Acceptance and commitment therapy |
|  | Relaxation training – e.g.) yoga, diaphragmatic breathing |
|  | Music therapy, Aromatherapy, Horticultural therapy, Food therapy |
| Health promotion | Education of nutrition and diet for cancer survivor |
|  | Education of cardiotoxicity and cardiovascular disease prevention |
|  | Education of vaccination for cancer survivor |
|  | Education of chronic disease management |
|  | Education of importance of secondary cancer screening |
| Social welfare | Providing information on social welfare system for cancer survivors (e.g.) support for economic problems, return to work) and linking the social welfare services |

This is a list of cancer survivor support programs. Each center operated some of these programs according to the center conditions.
